# Treatment strategies for breast cancer brain metastases

**DOI:** 10.1038/s41416-020-01175-y

**Published:** 2020-11-30

**Authors:** Caroline Bailleux, Lauriane Eberst, Thomas Bachelot

**Affiliations:** 1 Department of Medical Oncology, Centre Antoine Lacassagne, 33 avenue Valombrose, 06100 Nice, France; 2 Department of Medical Oncology, Institut de Cancérologie Strasbourg Europe, 17 rue Albert Calmette, 67200 Strasbourg, France; 3 Department of Medical Oncology, Centre Leon Berard, 28 rue Laënnec, 69373 Lyon, France

**Keywords:** Breast cancer, Metastasis

## Abstract

Brain metastases from breast cancer (BCBM) constitute the second most common cause of brain metastasis (BM), and the incidence of these frequently lethal lesions is currently increasing, following better systemic treatment. Patients with ER-negative and HER2-positive metastatic breast cancer (BC) are the most likely to develop BM, but if this diagnosis remains associated with a worse prognosis, long survival is now common for patients with HER2-positive BC. BCBM represents a therapeutic challenge that needs a coordinated treatment strategy along international guidelines. Surgery has always to be considered when feasible. It is now well established that stereotaxic radiosurgery allows for equivalent control and less-cognitive toxicities than whole-brain radiation therapy, which should be delayed as much as possible. Medical treatment for BCBM is currently a rapidly evolving field. It has been shown that the blood–brain barrier (BBB) is often impaired in macroscopic BM, and several chemotherapy regimens, antibody–drug conjugates and tyrosine-kinase inhibitors have been shown to be active on BCBM and can be part of the global treatment strategy. This paper provides an overview of the therapeutic option for BCBM that is currently available and outlines potential new approaches for tackling these deadly secondary tumours.

## Background

After lung cancer, metastatic breast cancer (MBC) is the second most common cause of brain metastases (BM) among solid malignancies.^1^ In a 2019 retrospective assessment of the metastatic breast cancer (MBC) cohort from the ongoing Epidemiological Strategy and Medical Economics (ESME) research programme, the risk of developing BM is estimated to be as high as 25% among patients with advanced breast cancer (BC), with a median time of BM occurrence 2–3 years after the initial BC diagnosis.^2^

**Fig. 1:**
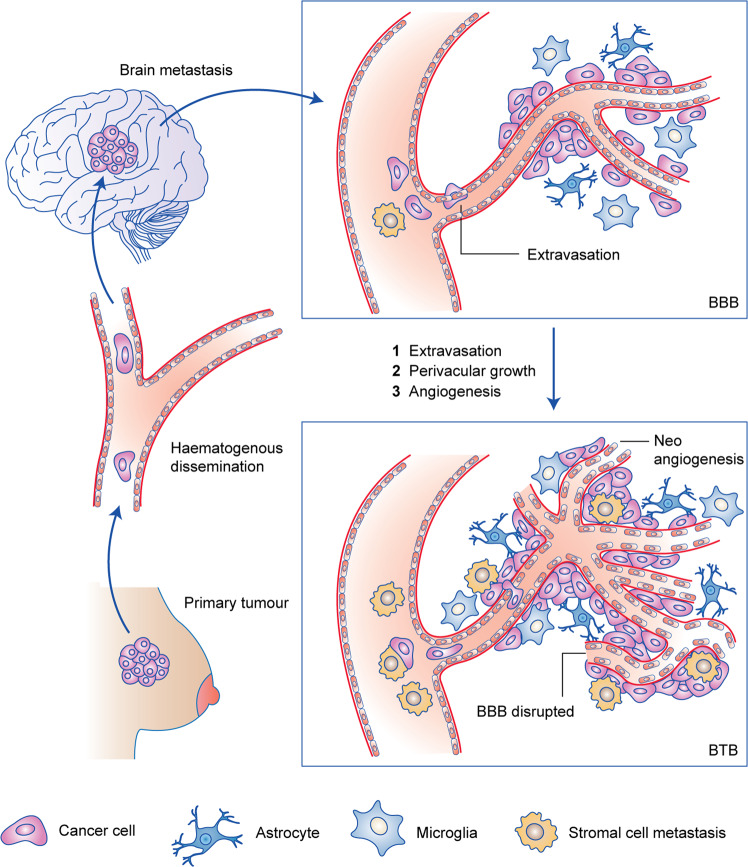
BMBC history. Following haematogenous dissemination, breast cancer cells invade the brain tissue by extravasation. Then they experience perivascular growth with no need of neoangiogenesis and the blood–brain barrier (BBB) is still intact. To reach macroscopic size, the new metastasis needs to stimulate neoangiogenesis. At this point, the BBB may be disrupted and is referred as the blood–tumour barrier (BTB). This Figure has been adapted from Eicher et al.^29^.

What is the history of BCBM? The main hypothesis is that cancer cells from breast parenchyma must undergo epithelial-to-mesenchymal transition (EMT) to enter the bloodstream, survive haematological diffusion and implant into the CNS after extravasation and a further step of reverse mesenchymal-to-epithelial transition (MET). Thus, cancer cells metastasising to the brain must possess a distinct set of adaptations to develop effectively in this unique environment with the acquisition of several fundamental characteristics to cross the blood–brain barrier (BBB), proliferate perivascularly and begin neoangiogenesis until the creation of a brain tumour barrier (BTB). Disruption of the BBB can also be promoted by radiotherapy and a BBB disruptor (cf infra) (Fig. 1).

The microenvironment of the brain, with its unique cell types, anatomical structures, metabolic constraints and immune environment, differs radically from the microenvironments of extracranial lesions, imposing a distinct selective pressure. Each metastatic localisation has particular characteristics in favour or not of the metastatic process, but patients with MBC are highly susceptible to bone, liver or lung metastasis as well, showing the high level of selection and advancement of metastatic disease when cancer cells are able to penetrate the central nervous system (CNS). Furthermore, breast cancer cells tend to metastasise preferentially in the cerebellum, suggesting that differences in the surface properties of tumour cells and vascular endothelium should exist even within brain areas.^3,4^

The microenvironment of the brain is characterised by unique anatomical structures,^5^ cell types, metabolic pathways^6^ and local immune environments^7^ in comparison with the microenvironment of the breast and of other extracranial lesions,^8^ which means that cells metastasising to the brain must possess a distinct set of characteristics to not only cross the BBB but also to grow effectively in this unique environment.^9^ Shifts in the levels of hormone receptors and the human epidermal growth factor receptor 2 (HER2) status have been reported in intracranial metastases compared with primary breast cancer cells and extracranial metastases.^10–12^

The branched evolution and selection pressure during blood dissemination and brain implantation of metastasis could explain these shifts. As a direct consequence, the usual treatment may not be adapted to the new-known subtype and may be completely useless against BCBM. BM was also found to harbour genetic changes that were not detected in the primary tumour, including actionable mutations that were eligible for targeted therapy although, beyond HER2 and oestrogen receptor (ER) status, the therapeutic implication(s) of such mutations remain to be established.^13^

Current therapeutic options for patients with BCBM include surgical resection, stereotactic radiosurgery (SRS), whole-brain radiation therapy (WBRT), chemotherapy and targeted therapy. Conventional systemic therapy has been considered a challenge for BCBM treatment as complex molecules are theoretically not capable of crossing the BBB. Nevertheless, this concept has been challenged as the results from some clinical trials indicate that systemic therapies can be effective for the treatment of BM, as discussed in this paper.

We begin this review by providing an epidemiological overview of BCBM before outlining the potential influence of the BBB on drug delivery and efficacy. After a full description of the current therapeutic strategy regarding surgery, radiotherapy and systemic treatments in the main breast cancer subtypes, we present the possible new avenues for future research. A selection of ongoing clinical trials discussed throughout this review, the results of which could change practices in the future, is detailed in Table 1.

**Table 1:** Selection of ongoing clinical trials for BCBM.

Treatment	Clinical trial number	Official title	Phase	Study population	Number of patients	Study intervention	Primary outcome
*Local treatment*							
Pre- vs post-operative SRS	NCT03741673	A Phase 3 trial of pre-operative stereotactic radiosurgery (SRS) vs post-operative SRS for brain metastases	3	≤ 4-cm BM for single fraction or ≤7 cm for multifraction therapy	86	Pre-operative SRS vs post-operative SRS	Leptomeningeal disease (LMD)-free rate
Pre-operative single-fraction SRS	NCT03368625	A Phase 2 study of neoadjuvant stereotactic radiosurgery for large brain metastases	2 (one arm)	1–6 BM, 2–4 cm	30	One pre-operative fraction of SRS	Radiation toxicity
Fractionated SRS alone	NCT04061408	A Phase 2 pilot study of fractionated stereotactic radiotherapy (FSRT) in breast cancer patients with 1–10 brain metastases	2 (one arm)	1–10 BM HER2^+^	170	3–5 fractions of 8 Gy	Intracranial local tumour control rate
WBRT alone	NCT03075072	Whole-brain radiation vs stereotactic radiation (SRS) in patients with 5–20 brain metastases: a Phase 3, randomised clinical trial	3	5–20 BM	196	WBRT vs SRS	Quality of life
*Chemotherapy agents*							
Etirinotecan pegol	NCT02915744	A Phase 3 open-label, randomised, multicenter study of NKTR-102 vs treatment of physician’s choice (TPC) in patients with metastatic breast cancer who have stable brain metastases and have been previously treated with an anthracycline, a taxane and capecitabine	3	Non-progressing BM	178	NKTR-102 vs TPC (eribuline, ixabepilone, vinorelbine, gemcitabine, docetaxel and (nab)-paclitaxel)	OS
Nal-IRI (MM-398)	NCT03328884	Multicenter open-label, Phase 2 trial, to evaluate the efficacy and safety of nal-iri for progressing brain metastases in patients with HER2-negative breast cancer (the phenomenal study)	2 (one arm)	HER2^+^ excluded with BM	63	Nal-IRI 60 mg/m^2^ on D1 of a 14-day cycle	CNS ORR
*New-generation TKIs*							
Neratinib + T-DM1	NCT01494662	A Phase 2 trial of HKI-272 (neratinib), neratinib and capecitabine and ado-trastuzumab–emtansine for patients HER2-positive breast cancer and brain metastases	2 (four cohorts)	HER2^+^ with BM	168	* Previously untreated * Progressive BM * Progressive BM after T-DM1	ORR (CNS ORR)
Tucatinib + T-DM1	NCT03975647	Randomised, double-blind, Phase 3 study of tucatinib or placebo in combination with ado-trastuzumab–emtansine (T-DM1) for subjects with unresectable locally advanced or metastatic HER2 + breast cancer (HER2CLIMB-02)	3	HER2^+^ with or without BM	460	Tucatinib + TDM1 vs placebo + TDM1	PFS
*New drugs*							
PARPi							
Veliparib + cisplatin	NCT02595905	Phase 2 randomised placebo-controlled trial of cisplatin with or without ABT-888 (veliparib) in metastatic triple-negative breast cancer and/or BRCA-mutation-associated breast cancer, with or without brain metastases	2 (randomised, two arms)	TN and/or BRCA-mutated mBC with or without BM	333	Cisplatin + placebo vs cisplatin + veliparib	PFS
*Immuno-oncology therapy*							
SHR-1316	NCT04303988	A prospective, single-arm, single-center, multi-cohort Phase 2 clinical study of HER2-positive and triple-negative breast cancer brain metastases	2 (HR^−^/HER2^−^ and HR^+^/HER2^+^ )	HR^−^/HER2^−^ with BM	59	SHR-1316 or bevacizumab or platin	CNS ORR
Nivolumab + SRS	NCT03807765	Phase 1b study of stereotactic radiation and nivolumab in the management of metastatic breast cancer brain metastases	1b	1–10 BM largest ≤ 4 cm	12	Nivolumab + SRS the week after initial dose of Nivolumab	Number of patients experiencing DLTs
Atezolizumab + SRS	NCT03483012	A Phase 2 study of atezolizumab in combination with stereotactic radiation for patients with triple-negative breast cancer and brain metastasis	2	TN BCBM largest ≤ 3 cm	45	Atezolizumab + SRS within 14 days after MRI	CNS-PFS
Pembrolizumab + SRS	NCT03449238	Pembrolizumab and stereotactic radiosurgery (Srs) of selected brain metastases in breast cancer patients	1/2 (one arm)	2 BM minimum largest ≤ 4 cm	41	Pembrolizumab infused one day before SRS	Tumour-response for non-irradiated BM at 8 weeks
*Intrathecal trastuzumab*							
Weekly dose (French study)	NCT01373710	Phase 1/2 study of safety and efficacy of intrathecal trastuzumab administration in metastatic HER2-positive breast cancer patients developing carcinomatous meningitis	1/2	HER2^+^ carcinomatous meningitis	34	Trastuzumab weekly Phase 2 dose: 150 mg	Weekly MTD
Twice-weekly dose (US study)	NCT01325207	Phase 1/2 dose escalation trial to assess safety of intrathecal trastuzumab for the treatment of leptomeningeal metastases in HER2-positive breast cancer	1/2	HER2^+^ leptomeningeal metastases	34	10 mg or 20 mg or 30 mg or 40 mg twice-weekly for 4 weeks, then once-weekly 4 weeks then every 2 weeks	Number of DLTs
*High-dose trastuzumab*							
Intra-arterial cerebral infusion	NCT02571530	Phase 1 trial of super-selective intra-arterial cerebral infusion of trastuzumab after blood–brain barrier disruption for the treatment of cerebral metastases of HER2/Neu positive breast cancer	1	HER2^+^ with BM	48	Intra-arterial cerebral infusion of trastuzumab	MTD
*Blood–brain barrier disruption*							
ExAblate Neuro System	NCT03714243	A study to evaluate the safety and feasibility of blood–brain barrier disruption using MRI-guided focused ultrasound in the treatment of HER2-positive breast cancer brain metastases	Not applicable	HER2^+^ with BM	10	ExAblate Model 4000 type-2 temporarily	Adverse events
*Targeted therapy*							
Entrectinib or abemaciclib or GDC-0084	NCT03994796	Genomically-guided treatment trial in brain metastases	2 (three arms)	CDK or PI3K or NTRK/ROS1 mutation with BM	150	Arm1: Abemaciclib Arm2: GDC-0084 Arm3: Entrectinib	CNS ORR
Palbociclib + trastuzumab	NCT02774681	A Phase 2 single-arm study of palbociclib in patients with metastatic HER2-positive breast cancer with brain metastasis	2 (one arm)	HER2^+^ with BM	12	Palbociclib + trastuzumab IV	CNS radiographic response rate
Palbociclib + trastuzumab + lapatinib + fuvelstrant	NCT04334330	Palbociclib, trastuzumab, lapatinib and fulvestrant treatment in patients with brain metastasis from ER positive, HER2 positive breast cancer: a multicenter, prospective study in China	2 (one arm)	HER2^+^/ER^+^ with BM	48	Palbociclib + trastuzumab + lapatinib + fuvelstrant	CNS ORR
Tucatinib + abemaciclib + trastuzumab + AI	NCT03846583	Phase 1b trial of tucatinib in combination with abemaciclib and trastuzumab for patients with HER2-positive metastatic breast cancer	1b	ER^+^/HER2^+^ with or without BM	53	Tucatinib + abemaciclib + trastuzumab + AI	MTD
Afatinib	NCT02423525	A Phase 1 dose escalation and central nervous system (CNS) pharmacokinetic study of the ErbB family inhibitor afatinib in patients with recurrent or progressive brain cancer	1	Cohort f: BM Cohort h: leptomeningeal metastases	24	Afatinib: 80 mg/4d or 120 mg/4d or 180 mg/4d or 280 mg/7d	Rate of DLTs of pulsatile afatinib
Afatinib	NCT02768337	Cambridge brain mets trial 1: a proof-of-principle Phase 1b/randomised Phase 2 study of afatinib penetration into cerebral metastases for patients undergoing neurosurgical resection, both with and without prior low-dose, targeted radiotherapy	1b/2 (two arms randomised)	Operable BCBM	70	Afatinib + /− radiation Arm 1: no radiotherapy Arm 2: 2 Gy Arm 3: 4 Gy	Ratio of fatinib concentration in resected BM vs plasma
Afatinib + TDM1	NCT04158947	A randomised study of HER2-positive breast cancer patients with active refractory brain metastases treated with afatinib in combination with T-DM1 vs T-DM1 alone	1/2 (two arms randomised)	HER2^+^ with BM	130	Afatinib + TDM1 vs TDM1	Safety and tolerability of TDM1 and Afatinib RP2D
Pyrotinib + vinorelbine	NCT03933982	Pyrotinib plus vinorelbine in patients with brain metastases from HER2-positive metastatic breast cancer: a prospective, single-arm, open-label study	2 (one arm)	HER2^+^ with BM	30	Pyrotinib daily + vinorelbine weekly	CNS ORR
Pyrotinib + temozolomide	NCT04303988	A prospective, single-arm, single-center, multi-cohort Phase 2 clinical study of HER2-positive and triple-negative breast cancer brain metastases	2 (HR^−^/HER2^−^ and HR^+^/HER2^+ ^)	HR^+^/HER2^+^ with BM	59	Pyrotinib + temozolomide IV	CNS ORR
Pyrotinib + capecitabine	NCT03691051	Pyrotinib plus capecitabine in patients with brain metastases from HER2-positive metastatic breast cancer: a single-arm, open-label, ahead study	2	HER2^+^ with BM	102	Pyrotinib + capecitabine	intracranial-lesion-ORR
Sorafenib + WBRT	NCT01724606	Whole-brain radiotherapy (WBRT) with sorafenib for breast cancer brain metastases (BCBM): a Phase 1 study	1	New and/or progressive BM WBRT indication	21	Sorafenib 200 mg, 400 mg or 600 mg + WBRT	MTD
AZD1390 + WBRT	NCT03423628	A Phase 1, multicentre study to assess the safety, tolerability, and pharmacokinetics of ascending doses of AZD1390 in combination with radiation therapy in patients with glioblastoma multiforme and brain metastases from solid tumours	1	Arm B: BCBM eligible	132	1–3 cycles of AZD1390 + WBRT or PBRT	Incidence of DLTs
GDC-0084 + trastuzumab	NCT03765983	Phase 2 trial of GDC-0084 in combination with trastuzumab for patients with HER2-positive breast cancer brain metastases	2	HER2^+^ new and/or progressive BM	47	GDC-0084 + trastuzumab	CNS ORR
*MRI screening*							
MRI every 4 months	NCT03881605	Routine MRI screening vs symptom-directed surveillance for brain metastases among patients with triple-negative and HER2-positive metastatic breast cancer (MBC): a single-centre randomised pilot study	2 (randomised pilot study)	HER2^+ ^BCBM and triple-negative BCBM	50	MRI screening vs clinical symptoms every 4 months during 1 year	Proportion of eligible patients agree to enroll and complete protocol
Histology-adapted (Dana-Farber)	NCT04030507	Screening magnetic resonance imaging of the brain in patients with metastatic breast cancer managed with first/second-line chemotherapy or inflammatory breast cancer managed with definitive intent: a prospective study	2	BCBM cohort 1: TNBC cohorts 2 + 3: HR^+^/HER2^−^ and HER2^+^ cohort 4: inflammatory BC	214	Cohort 1: MRI/6 months Cohorts 2 + 3: MRI at diagnosis or not Cohort 4: MRI at diagnosis	Neurological quality of life at 12 months
Observational study	NCT03617341	Brain monitoring for high risk of brain metastases in metastatic breast cancer	Cohort	HER2^+^ and TNBC BCBM	200	MRI at diagnosis, first- and second-line treatment failure	BM incidence rates
Every 4 months vs every 12 months	NCT00398437	Role of gadolinium enhanced brain magnetic resonance in the follow-up of metastatic breast cancer patients overexpressing HER2 Neu. A randomised prospective study	Prospective randomised study	HER2^+^ No CNS metastases	96	MRI every 4 months vs every 12 months	Survival without neurological symptoms due to BM and/or leptomeningeal involvement

*OS* overall survival, *PFS* progression-free survival, *SRS* stereotactic radiosurgery, *ORR* overall response rate, *DLT* dose-limiting toxicity, *MTD* maximum tolerated dose, *CNS* central nervous system, *BM* brain metastasis, *TN* triple negative, *AI* aromatase inhibitor.

## BCBM epidemiology

### Incidence and prevalence

The incidence of BCBM has increased in in the last few years following improved survival rates of patients with MBC (particularly those with the HER2^+^ subtype) and increased detection of metastatic disease through advanced imaging techniques.^14,15^ Identified risk factors for BM are common risk and prognostic factors for metastatic disease: diagnosis of breast cancer before age 40,^16,17^ ER negativity,^16,17^ triple-negative status (ER^–^, progesterone receptor (PR)^−^ and HER2^−^),^18^ high histological grade,^19^ overexpression of HER2,^16,20^ presence of extracerebral metastases (pulmonary, hepatic and lymphatic), number of extracerebral metastatic sites and location of extracerebral metastases,^16,17^ short time from diagnosis of cancer to metastatic disease^21^ and elevated lactate dehydrogenase (LDH) levels prior to treatment.^22^

In the ESME cohort, 4118 patients with BM among 16,701 MBC patients were screened between 2008 and 2014 and followed up for a median of 42.8 months. The overall BM prevalence was 24.6% (7.2% at MBC diagnosis and 17.5% during follow-up). BM was diagnosed based on symptom occurrence in 70.7% of patients, and through systematic imaging examination in 29.3% of patients. Due to the lack of data demonstrating a clinical benefit, brain screening for patients with MBC is not currently recommended in the US National Comprehensive Cancer Network (NCCN) and European Society of Medical Oncology (ESMO) guidelines.^23^ This explains why for 70.7% of patients, symptoms lead to diagnostic brain imaging. The ESME cohort is a real-life cohort that illustrates the daily practice in French cancer centres and hospitals. The prevalence of BM varied according to initial histological subtype of BC, with increased BM risk for ductal carcinoma, Scarff–Bloom–Richardson (SBR) grade III, ER^+^/HER2^+^, ER^–^/HER2^+^ and triple-negative (ER^–^, PR^–^ and HER2^–^) subtypes. At initial BC diagnosis, BM was detected in 4.3% of ER^+^/HER2^–^, 9.2% of ER^+^/HER2^+^, 17% of ER^–^/HER2^+^ and 13% of triple-negative subtypes. Patients presented BM during their metastatic disease in 19% of ER^+^/HER2^–^, 34% of ER^+^/HER2^+^, 49% of ER^–^/HER2^+^ and 38% of triple-negative metastatic breast cancers.^2^ The development of BM in triple-negative BC patients frequently occurs with concurrent extracranial disease progression,^18^ in contrast with HER2-overexpressed BM, which often occurs with a stable extracranial status.^24^ BM-free survival (BM-FS) rates at 5 years were 68%, 50% and 30% for ER^+^/HER2^–^, ER^+^/HER2^+^ and ER^–^ subtypes, respectively. A direct correlation between the time from first relapse and the incidence of BM existed for all three subgroups. For patients with ER^–^ disease (HER^–^ or HER2^+^), the prevalence of BM is 70% 5 years after metastatic diagnosis.

### Survival and prognosis

During a 30-month follow-up, the median overall survival (OS) after BM diagnosis was 7.9 months. OS was independently associated with subtypes: the median OS after BM diagnosis was 18.9 months for ER^+^/HER2^+^, 13.1 months for ER^–^/HER2^+^, 7.1 months for ER^+^/HER2^–^ and 4.4 months for ER^–^/HER2^–^ (*P* < 0.0001).^2^ These differences in OS following BM diagnosis are mostly due to available and efficient systemic treatment—before the anti-HER2 therapy revolution, survival outcomes were comparable between patients with HER2^+^ and HER2^–^ MBC. In a retrospective study, Dawood et al. showed that patients with HER2^+^ disease and CNS metastases treated with the recombinant humanised IgG1 monoclonal anti-HER2 antibody trastuzumab had better survival (11.6 months) compared with patients with HER2^+^ disease who had never received trastuzumab (6.1 months; hazard ratio (HR) for death 1.34, 95% confidence interval (CI) 0.78–2.30, *P* = 0.28) and patients with HER2^–^ breast cancer (6.3 months, HR for death 1.66, 95% CI 1.31–2.12, *P* < 0.0001).^24^

The poorer prognosis of patients with ER^+^/HER2^–^ disease compared with patients with HER2^+^ disease is most likely due to the fact that BM is a late event in metastatic disease evolution in this subtype. Factors identified as conferring a good prognosis at the time of diagnosis of BM are young age (<60 years), good ECOG-performance status, molecular subtype (ER^+^ and/or HER2^+^), single BM and controlled systemic disease.^25,26^

## The blood–brain barrier

As well as presenting a potential barrier to metastasising cells, the BBB can hinder the delivery of systemic therapies into brain tumours, thus decreasing intracranial response rates to treatment^27^ (Box 1).

However, with the growth of new, abnormal, vessels during tumour progression, the BBB can become disrupted and is referred to as the blood–tumour barrier (BTB).^28,29^ The BTB is more leaky than the BBB and heterogeneous, including in such permeability, which results in the uneven distribution of drugs in mouse models of brain metastasis.^28^ These characteristics could explain the discordant results from preclinical studies, some of which clearly favour a lower concentration of drug in experimental BM, while others have shown that some chemotherapy drugs can penetrate experimental BM as efficiently as they can for extracerebral metastatic disease.^27,28,30–32^ Furthermore, pharmacokinetics particularity might not be the only barrier to drug efficacy in BM. Preclinical studies have shown that trastuzumab penetrates experimental BM as efficiently as it does extracerebrally localised metastases, but nevertheless shows decreased activity in the brain.^32^ These results have led some authors to reconsider whether mechanisms beyond inadequate drug penetration across the BBB, such as brain-specific drug resistance, might be operative in BM.^33^ Translational clinical diffusion studies of patients with CNS metastatic disease have also shown that chemotherapy and trastuzumab can accumulate in BM. Fine et al. report that patients who receive an infusion of paclitaxel prior to BM surgery show a significant concentration of taxane in the metastatic tissue, particularly at the centre of the metastases:^34^ 2507 ng/gm tumour for an estimated therapeutic level of >1000 ng/gm.^27^ Dijkers et al. have shown that [^89^Zr]-trastuzumab can be detected in BM at the same median concentration than in bone metastasis of patients with HER2^+^ MBC.^35^ However, the results of Lockman et al. and Dijkers et al. in humans revealed a large variability of concentrations in BMs. Taken together, these studies show that the BTB is more permeable to chemotherapy than the BBB, at least in the case of macrometastatic disease with established neovascularisation.^28^ The difference between these two entities (BBB and BTB) might explain the observed activity of most medical treatment on established BM (see below).

However, BBB-generated ‘sanctuary’ might be more relevant with regard to micrometastatic deposits in the adjuvant setting and for meningeal carcinomatosis. Neovascularisation plays a key role in the BBB disruption. It occurs in response to central hypoxia in large nodular metastases. Micrometastases and meningeal carcinomatosis are therefore less neovascularised and therefore their BBB is theoretically less permeable than macrometastases’ one. Indeed, large positive adjuvant trials of HER2-targeted therapy repeatedly failed to show any significant decrease in CNS relapse in the experimental arm compared with the control arm.^36,37^ Furthermore, in patients receiving standard intravenous trastuzumab, pharmacokinetic analysis showed infra-therapeutic concentrations in the lateral cephalometric radiograph.

Box 1 (see Fig. 1) Blood–brain barrier (BBB)

- Highly selective semipermeable border of endothelial cells that prevents *non-selectively* crossing from blood into the extracellular fluid of the central nervous system.
- Selectivity is due to the tight junctions between the endothelial cells of brain capillaries.
- Astrocyte cell projections provide biochemical support to endothelial cells.
- Microglial cells, primary immune cells of the CNS, involved in immune response and homoeostasis by scavenging, phagocytosis and extracellular signalling.
- Mesenchymal stroma cells protect the blood–brain barrier.

Blood–tumour barrier (BTB)

- Highly heterogeneous with non-uniform permeability and active efflux of molecules.
- Tumoral invasion, perivascular growth and neoangiogenesis disrupted BBB creating BTB.
- Epithelial cells and tight junctions are disconnected and allow non-selectively crossing from blood into the extracellular fluid.
- Tumoral mesenchymal stroma cells could metastasise with cancer cells and support tumoral development.

## Current treatment options for BMBC

Current therapeutic options for BM include both local (surgery and radiotherapy) and systemic treatments (chemotherapy, therapeutic antibodies and tyrosine-kinase inhibitors) or a combination of several modalities.^38–40^ Regardless of the initial BC subtype, if the estimated life expectancy is greater than 3 months and extra-CNS disease is controlled, it is recommended that cases of up to 10 BM be systematically discussed by multidisciplinary staff. If the estimated life expectancy is less than 3 months, appropriate supportive care, whole-brain radiotherapy (WBRT) or exclusive systemic treatment will be proposed.^41^ Given that not only breast cancer but other cancer types were included in the radiotherapy studies detailed in the following paragraphs, the results cannot discard the influence of this variable in the results.

### Surgery followed by radiotherapy

The use of surgery is most often reserved for patients with good performance status, few lesions or large symptomatic lesions (≥3 cm). Surgery followed by radiation therapy has been shown to improve OS and symptom control vs radiation therapy alone.^42,43^ Patchell et al. demonstrated that, in patients with a single BM (comprising 9.5% of cases of BMBC), WBRT after complete surgical resection reduced the rate of recurrence at both the initial metastatic site (10% vs 46%, *P* < 0.001) and other brain sites (14% vs 37%, *P* < 0.01), and reduced death due to intracranial progression (14% vs 44%, *P* = 0.003). However, OS was similar between the WBRT and the control arm.^44^ In addition, WBRT has short- and long-term toxicities, including neurocognitive side effects and decreased quality of life.^45,46^

Two randomised clinical trials compared post-operative WBRT to post-operative stereotactic radiosurgery (SRS), a precise form of radiotherapy that delivers highly conformal high-dose radiation to restricted areas to kill small groups of cells with minimal damage to the surrounding normal tissues.^47,48^ One of these studies included patients with up to three lesions. In both studies, local control was equivalent or better with SRS and there was a lower risk of cognitive impairment; no differences in OS were seen between the radiotherapeutic approaches. Therefore, after surgical resection, the use of SRS is the recommended option whenever feasible.^47,48^

### Pre-operative SRS

In a multi-institutional retrospective analysis with 180 patients receiving pre-operative SRS or postoperative SRS, Patel et al. demonstrated a significantly lower rate of symptomatic radionecrosis (16.4% vs 4.9%) and leptomeningeal disease (cancer in the leptomeninges, 16.6% vs 3.2%) using the neoadjuvant approach, but similar rates of local recurrence, distant brain recurrence and OS occurred between the two arms (37.5% BMBC).^49^ A two-arm trial in which patients are randomised to receive SRS before or after surgical removal aims to investigate whether 1-year leptomeningeal disease-free survival will be increased using the neoadjuvant approach (NCT03741673). The neoadjuvant SRS (NaSRS) non-randomised Phase 2 study is investigating the outcome of patients with 1–6 BCBMs treated with a single fraction of SRS before surgical removal of the metastases (NCT03368625).

### SRS alone

When surgical resection is not feasible, SRS alone is the recommended approach. Given the short- and long-term neurological toxicities associated with the use of WBRT, its use should be delayed as long as possible in favour of SRS. A retrospective trial carried out in Japan has shown that patients with up to ten metastases can be safely treated using SRS without increased toxicities (20.6% BMBC). Patients with 2–4 BM and patients with 5–10 BM had similar OS (10.8 months in both arms). Each treatment group had excellent local control (89% vs 90%) and required low rates of salvage WBRT (10 vs 8%). Two-year neurological death, new brain-lesion incidence and neurocognitive outcome were also similar between the two groups. However, the risk of leptomeningeal dissemination was significantly higher in the 5–10 BM arm than in the 2–4 BM arm (22% vs 13%, respectively).^50,51^ As there are more metastases, either the diffusion process is more advanced or the cancer cell seeds have a greater affinity for the brain tissue. Consequently, the probability of minimal initial carcinomatous meningitis or secondary diffusion is greater.

Grandhi et al. demonstrated a high rate of local control in patients with ≥10 BM receiving SRS alone (24.6% BMBC) but observed a median OS of only 4 months.^52^ A Phase 2 trial in China examined whether fractionated doses of SRS were safer (reducing necrosis) and more effective (increasing local control) than single-dose SRS for patients with 1–10 BM (NCT04061408). In any case, the global volume of BM, rather than the total number of BM, seemed to be an important factor to take into account.^50^ In patients with up to ten metastases and a volume of less than or equal to 30 cc, SRS is the recommended treatment option.^41^

### WBRT alone

The use of WBRT alone is indicated only in patients with more than ten BM for whom local treatment is not appropriate and in patients with new lesions on which additional SRS cannot be performed.^41^ Repeat SRS twice or more time on progressive BM is an increasingly used therapeutic option, although little data are available to support this practice. The tumour control following repeat SRS for locally recurring metastatic brain tumours after a previous SRS is relatively lower than that for primary SRS. However, both low tumour volume and high-prescription radiation dose were significantly related to the tumour control following repeat SRS for these tumours after previous SRS, which is a general understanding of primary SRS for metastatic brain tumours.^53^ In asymptomatic patients, a systemic therapy approach, based on the BC subtype, might be preferred. In the absence of cerebral symptomatology, the urgency is to systemic control and the best treatment for brain metastasis remains the treatment of cancer. WBRT should be limited to symptomatic patients without feasible systemic therapy options and with an urgent need of symptom relief. In particular for HER2^+^ BCBM patients, the late-onset toxicities of WBRT should be weighed up against the favourable prognosis as indicated by different prognostic scores.^54^ It remains unknown whether SRS improves the quality of life of patients with 5–20 BM relative to WBRT—SRS avoids whole-brain radiation, but the potential risk of recurrence is increased. A randomised trial that compares WBRT to SRS for patients with 5–20 metastases who have received no prior radiation is ongoing, with quality of life as the primary endpoint (NCT03075072).

A notable development in WBRT is the use of intensity-modulated radiotherapy techniques—termed hippocampal avoidance WBRT (HA-WBRT)—that avoid hippocampal neural stem cells, which are essential for new memory formation.^55^ Encouraging data on toxicity were reported in Phase 2 studies of HA-WBRT when compared with the historical control, WBRT without hippocampal avoidance.^56^ A large, randomised clinical trial of 518 patients (18.5% BMBC) assigned to HA-WBRT or WBRT revealed no difference in clinical progression between the two arms, but the HA-WBRT group experienced a significantly lower risk of cognitive function deterioration (HR, 0.74, *P* = 0.02). This result favours the use of HA-WBRT over classical WBRT.^57^ When SRS is not possible, HA-WBRT is the recommended option.^57^

### Chemotherapy agents

For systemic therapies, various drugs, including older chemotherapy agents such as capecitabine, cyclophosphamide, 5-fluorouracil, methotrexate, vincristine, cisplatin, etoposide, vinorelbine and gemcitabine, have shown activity in the treatment of BM, with an objective response rate (ORR) of over 30%, a median duration of neurological remission for responder patients of up to 30 weeks and a median OS of up to 31 weeks, similar to the systemic response rates usually observed similar to the systemic response rates commonly observed with the same drug molecules.^34,58,59^ Combinations such as cisplatin–etoposide, capecitabine–temozolomide and cisplatin–temozolomide have been analysed in retrospective Phase 1 and Phase 2 studies, with an ORR of up to 40%, a median progression-free survival (PFS) of up to 2.9 months and a median OS of up to 5.5 months.^59–61^ However, the results of these slightly older studies might be affected by incomplete diagnostic imaging (cranial CT scan instead of MRI, for example) and non-standard chemotherapy regimens. Consequently, the evidence for the efficacy of chemotherapeutic agents for patients with BCBM remains limited. Furthermore, owing to the limited number of patients with BCBM, none of these protocols has been approved for the treatment of this indication. Furthermore, the PFS and OS figures are dismal, and traditional chemotherapy alone cannot be considered to be a satisfactory treatment for BCBM. Nevertheless, these studies do support the fact that the BTB is at least partially permeable to systemic treatment.

New chemotherapy agents, such as third-generation taxanes, are in development for the specific indication of BCBM. Several polymeric conjugates of irinotecan and SN-38 (the active metabolite of irinotecan) are also being investigated. These include etirinotecan pegol and MM-398.^62^ Etirinotecan pegol is a long-acting derivative of irinotecan that prolongs exposure to SN-38 while reducing its toxicity. In a predefined sub-study of the BEACON study, in which 67 patients with BCBM were randomised to etirinotecan pegol or a treatment of physician’s choice—eribulin, vinorelbine, gemcitabine, nab-paclitaxel, paclitaxel, ixabepilone or docetaxel—longer survival was observed in the etirinotecan pegol group (10.0 months vs 4.8 months, HR 0.51, *P* < 0.01).^63^ This molecule is currently being evaluated against the treatment of the investigator’s choice in a randomised trial in patients with stable brain dissemination (ATTAIN Phase 3 NCT02915744). Another Phase 2 international trial is currently evaluating the efficiency and safety of MM-398, a nanoliposomal irinotecan thought to penetrate the BBB, in patients with HER2^–^ MBC (The Phenomenal Study, NCT03328884).

### Trastuzumab-based regimens for patients with HER2^+^ BM

To date, evidence for the direct efficacy of HER2-targeting monoclonal antibodies trastuzumab, trastuzumab–emtansine (T-DM1) and pertuzumab on BMBC is based on the retrospective subgroup analysis of clinical trials or on small cohorts. Nevertheless, evidence mostly argues in favour of the clinical efficacy of these molecules on BM.

Park et al.^64^ demonstrated that BC patients receiving trastuzumab had a significantly longer median time to BCBM (15 months vs 10 months, *P* = 0.035) and median time to death (14.9 vs 4.0 months, *P* = 0.0005) than patients who were not treated with trastuzumab. Similarly, Rostami et al.^65^ also demonstrated that the mean survival of patients with HER2^+^ BCBM was prolonged when treated with trastuzumab (17.5 vs 11 months). In a retrospective study, Dawood et al.^24^ showed that patients with HER2^+^ disease treated with trastuzumab had longer median time to CNS metastasis (13.1 months) compared with patients with HER2^+^ disease who had never received trastuzumab (2.1 months, HR 2.13, 95% CI 1.51–3.00, *P* = 0.28) and patients with HER2^–^ breast cancer (8.9 months, HR 1.5, 95% CI 1.15–1.95, *P* < 0.0001).

These studies show a correlation between the use of trastuzumab and the development of less aggressive BM. However, it is impossible from these results to conclude whether trastuzumab had a direct effect on BM or an indirect one (perhaps fewer secondary BM was formed following new systemic lesions).

Trastuzumab–emtansine (T-DM1) is an antibody–drug conjugate that has been approved for the second-line treatment of HER2^+^ MBC after the failure of trastuzumab and pertuzumab, following the results of the randomised Phase 3 EMILIA trial.^66^ T-DM1 was associated with a statistical and clinically meaningful improvement of PFS and OS (HR for OS = 0,68, *P* < 0,001) compared with a combination of the tyrosine-kinase inhibitor (TKI) lapatinib and capecitabine. An exploratory analysis of second-line T-DM1 focusing on patients with treated and asymptomatic BM at baseline showed improved OS in the T-DM1 group over the lapatinib–capecitabine group (26.8 vs 12.9 months, HR = 0.38, *P* = 0.008). Otherwise, the incidence of BM progression was similar between the T-DM1 arm and the capecitabine–lapatinib arm.^67^ Bartsch et al. investigated the intracranial response rates to T-DM1 treatment in BCBM patients. T-DM1 was administered at a dose of 3.6 mg once every 3 weeks as a primary systemic therapy for BM or upon documented CNS progression after initial local treatment. At 8.5 months median follow-up, intracranial PFS was 5 months and median OS from initiation of T-DM1 was not reached.^68^ Direct efficacy of T-DM1 on BM has also been shown in several retrospective analyses.^69^ A subgroup analysis of 398 patients with BM included in the KAMILLA single-arm open-label, Phase 3b study published in 2020, confirms the efficacy and safety of T-DM1 in this situation, with a BM response rate of 21%, a median PFS of 6 months and a median OS of 19 months.^70^

#### Pertuzumab–trastuzumab combination for patients with HER2^+^ BM

Pertuzumab is a recombinant humanised monoclonal antibody targeting the dimerisation domain II of HER2 that was approved for the first-line treatment of patients with HER2^+^ MBC following the randomised Phase 3 placebo-controlled CLEOPATRA trial. This study showed that the combination of pertuzumab with trastuzumab and a taxane was superior to the standard trastuzumab plus taxane combination (HR for PFS 0.68, *P* < 0.001; HR for OS: 0.68, *P* < 0.001).^71^ Although the patients reportedly did not have BM at diagnosis, disease relapse occurred in the brain in 13% of patients. As these latter patients did not develop systemic disease before brain progression, it is likely that this progression actually resulted from pre-existing small BM that was undetected at diagnosis. In this subpopulation, the median BM-PFS increased from 11.9 to 15 months with the addition of pertuzumab (HR = 0.58, *P* = 0.0049).^72^ Furthermore, median OS tended to be longer in the pertuzumab arm (34.4 months) compared with the control arm (26.3 months). These results suggest that the pertuzumab–trastuzumab and taxane combination shows comparable activity on BM and systemic disease. Nevertheless, a specific prospective study is warranted to confirm this hypothesis.

### First-generation TKIs for the treatment of HER2^+^ BM

Small-molecule tyrosine-kinase inhibitors (TKIs) are promising agents for the treatment of Her2^+^ BCBM as they cross the BBB and can block several receptors of the ErbB2 family. Lapatinib, a first-generation TKI, is a dual inhibitor of HER1 and HER2 kinases. Used as a monotherapy, this agent has shown poor results for BM,^73^ but better results were obtained in combination with capecitabine. The lapatinib–capecitabine combination is associated with a brain-response rate of up to 38% after radiotherapy.^73–75^ In the LANDSCAPE trial, lapatinib–capecitabine given at the time of BM diagnosis led to a response rate of 66%; the median time to progression (TTP) was 5.5 months and the median time to brain radiotherapy was 8.3 months.^76^ These results are consistent with the efficacy of this therapeutic combination on extracerebral diseases such as BC,^77^ but remain modest in terms of clinical benefits. In this trial, half of the patients had asymptomatic BM, and therefore intracranial response rates might have been overestimated. However, the results indicate a better outcome for patients diagnosed at an early stage of intracranial disease and therefore raise the question of implementing a standard screening procedure in a high-risk population (discussed later). In 2011, Lin et al. published the results from a randomised Phase 2 trial comparing the efficacy and safety of lapatinib–capecitabine with lapatinib–topotecan in patients pretreated with trastuzumab and cranial radiotherapy for BCBM.^78^ This study was discontinued early because the lapatinib–topotecan arm was too toxic and no objective response was observed. However, the response rate in the lapatinib–capecitabine arm was 38%.

Despite these important results, it has not been shown that the lapatinib–capecitabine combination was superior to trastuzumab-based treatment for BM control—neither as a ‘preventive’ treatment for patients without BM at relapse in the first-line CEREBEL study comparing lapatinib–capecitabine with trastuzumab–capecitabine (3% vs 5%, *P* = 0.36),^79^ nor for established BM when compared with T-DM1 in the subgroup analysis of the EMILIA study.^67^ Notably, these results provide additional evidence that the BTB is generally permeable to common systemic treatment, resulting in comparable results on BM and systemic disease in most randomised trials.

### New-generation TKIs for the treatment of HER2^+^ BM

New TKIs with a specific BM endpoint, particularly neratinib, a new irreversible pan-HER TKI and tucatinib, a HER2-specific TKI,^80,81^ have been developed.

Neratinib has been tested in combination with capecitabine in patients with HER2^+^ BCBM.^82^ Of the 37 patients, 89%, 22% and 14% were previously treated with trastuzumab, T-DM1 and another investigational HER2-directed agent, respectively, and most had received previous radiotherapy (65% WBRT and 32% SRS) and several chemotherapy agents. Freedman et al. reported 18 partial responses, with a BM volumetric response of 49%, 6-month PFS of 38% and a median time-to-BM progression of 5.5 months. Fifty-one percent (51%) of patients experienced grade 3 toxicities, of which 32% were gastrointestinal events, mostly diarrhoea, requiring specific prophylactic management. This result was supported by the NALA -randomised second-/third-line trial, including 130 patients with non-progressive BM at study entry.^83^ The overall cumulative incidence of intervention for BM was reduced from 29.2% with lapatinib–capecitabine to 22.8% with neratinib–capecitabine (*P* = 0.043).^83^ Cohort 4 of the Phase 2 trial NCT01494662 will study the combination of neratinib and T-DM1 for patients with untreated or progressive HER2^+^ BCBM with or without prior treatment with T-DM1.

The activity of tucatinib on BM was a prespecified secondary endpoint of the large pivotal HER2CLIMB trial,^84^ which analysed the addition of tucatinib to trastuzumab plus capecitabine in patients with pretreated HER2^+^ MBC. Patients with BM could be included even if BM was not pretreated or if they were active after local treatment. For patients with a history of BM (291 patients), the risk of progression or death was reduced by 68% with the addition of tucatinib vs placebo (HR 0.32, *P* < 0.00001), with a median PFS of 9.9 months vs 4.2 months, respectively. Impressively, median OS was 18.1 months in the tucatinib arm vs 12.0 months in the placebo arm (HR, 0.58, 95% CI, 0.40–0.85, *P* = 0.005). In fact, the efficacy of tucatinib on this BM population is consistent with the efficacy of tucatinib on the whole population (HR for PFS 0.54, *P* < 0.001; HR for OS: 0.66, *P* = 0.005). The main toxicity was diarrhoea, with 12.9% of grade 3 in the tucatinib arm vs 8.6% in the placebo arm.^84^ This study is the largest randomised trial to date with a specific BM endpoint and consequently provides the highest levels of evidence regarding medical treatment of patients with HER2^+^ BCBM. Interestingly, it shows that, once again, the effects of medical treatment on the progression of brain disease are mostly equivalent to those observed in extracerebral diseases. The HER2CLIMB2 trial comparing T-DM1 associated with tucatinib vs T-DM1 alone is ongoing (NCT03975647).

### Treatment for patients with HER2^–^ BM

For the treatment of patients with ER^+^/HER2^–^ BCBM, some responses have been observed with tamoxifen,^85^ aromatase inhibitors^86,87^ and fulvestrant^88^, and therefore, in the absence of therapeutic alternatives, these low-toxicity treatments have a place in paucisymptomatic ER^+^ patients.

Studies assessing late-line agents that target cyclin-dependent kinases (CDKs) 4 and 6 have been published. In an open-label, Phase 2 trial, abemaciclib was proposed for the treatment of patients with ER^+^/HER2^−^ (Part A) or ER^+^/HER2^+^ (Part B) BCBM. Patients in Part B did not reach the primary endpoint, the investigator-assessed objective response rate (ORR). Patients in Part A (*n* = 58) had an intracranial ORR of 5.2%, an intracranial clinical benefit rate of 24% and a median PFS of 4.9 months with some long responses (*n* = 11). A subgroup of 8 patients in this study had surgical resection of BM while undergoing treatment, and it was shown in the resected samples that therapeutic levels of abemaciclib were reached in BM, demonstrating abemaciclib and its active metabolites penetrated the BBB.^89^

For patients with triple-negative BCBM, two chemotherapy regimens seem to show specific CNS activity: the anti-vascular endothelial growth factor agent bevacizumab plus paclitaxel in a small Phase 2 study (70% ORR but only 6 patients with triple-negative MBC)^90^ and the microtubule inhibitor eribulin in case reports.^91^ A Phase 2 trial presented at ASCO 2013 highlighted a combination of bevacizumab plus carboplatin in the treatment of BCBM.^92^ In this study, 38 patients were treated with bevacizumab plus carboplatin, and trastuzumab was added if the tumour was HER2^+^. The composite brain ORR was 63% and the global ORR was 45%. For these HER2^–^ patients, therefore, standard chemotherapy comprising capecitabine, eribulin or carboplatin plus bevacizumab can be used for progressive BM after local treatment.

## Potential new drugs for the treatment of BM

According to the ClinicalTrial.gov site (accessed May 2020), there are 108 studies on BCBM, of which 24 are recruiting to test new drugs, including poly-ADP ribose polymerase (PARP) inhibitors (4 studies), immuno-oncology therapy (4), CDK4/6 inhibitors (2), TKIs (7), phosphatidylinositol 3-kinase (PI3K) inhibitors (1), ATM inhibitors (1) and BBB disruptors (1).

### PARP inhibitors

Inhibitors of the enzyme PARP are approved for the treatment of germline *BRCA*-mutated MBC. In the Phase 3 EMBRACA trial of patients with *BRCA*-mutated advanced and/or MBC, 15% of patients in the talazoparib treatment arm had stable/treated CNS disease at baseline. In this subgroup analysis, the benefit in terms of PFS was even more significant than it was for patients without BM (HR 0.32, 95% CI 0.15–0.68 and HR 0.58, CI 95%, 0.43–0.78, respectively), suggesting the possibility of a CNS effect of the drug.^93^ In the OLYMPIAD study, the benefit of olaparib vs practitioner’s choice among patients with HER2^–^ germline *BRCA*-mutated BCBM appeared to be comparable with the benefit observed in the whole study population, for both PFS (8.3 months vs 2.8 months) and ORR (64.7% vs 20.0%). The study was not designed to detect differences between subgroups, with a small number of patients and insufficient power, and so the results should be interpreted with caution.^94^ In BROCADE 3 with veliparib, only 4% of patients had a history of CNS metastases; the evidence of efficacy on BCBMs is therefore limited.^95^

Veliparib is the first PARP inhibitor to be tested specifically in BCBM; it was used in combination with WBRT in a Phase 1 trial recruiting patients with BM from primary solid tumours.^96^ Median OS in the breast cancer group was 8 months (2.8–15.0 months) vs an OS of 4.9 months predicted by nomogram modelling. A nomogram represents the relations between three or more quantitative variables by means of several scales, arranged in such a way that the value of a variable can be found by a simple geometric construction, for example by drawing a straight line intersecting the other scales at the appropriate values. The main toxicities associated with this treatment approach were asthenia (30%) and nausea (20%), with no new toxicities observed.^96^ Additional studies with PARP inhibitors are ongoing: SWOG S1416 is a Phase 2 study of cisplatin with or without veliparib in patients with metastatic triple-negative breast cancer and/or *BRCA*-mutation-associated breast cancer, with or without brain metastases (NCT02595905).

### Immuno-oncology therapy

The development of immuno-oncology drugs for the treatment of BM was initially slow owing to the concept that the brain is ‘immunologically privileged’—that is, antigens present in the brain do not elicit an inflammatory immune response. However, there is some evidence that immune infiltrates are present in melanoma-derived BM,^97^ and clinical trials in metastatic melanoma patients reported comparable ORR and OS in patients with or without asymptomatic BM at inclusion treated with the immune-checkpoint inhibitors nivolumab (which targets the programmed death receptor-1 (PD-1)) and ipilimumab (which targets CTLA-4).^98^ Immuno-oncology therapy could therefore be effective against BM as it is against other metastatic sites.

Atezolizumab (an antibody that targets the PD-1 ligand, PD-L1) has been approved in combination with nab-paclitaxel for the treatment of triple-negative MBC. Subgroup analyses showed a benefit for patients without BM (HR 0.80, 95% CI 0.69–0.93) but not for patients with BM (HR 0.86, 95% CI 0.50–1.49). However, the HRs were similar, and the absence of statistical significance could be explained by the small population size with BM, as only 6.3% of the study population had a history of BM (61 patients).^99^ As with other tumour locations, if immunotherapy proves effective for MBC, patients with BCBM could also benefit from an intracerebral response. A Phase 2 study is underway to evaluate the efficacy and safety of a new anti-PD-L1 antibody (SHR-1316) in combination with cisplatin/carboplatin and bevacizumab for triple-negative BCBM. The primary endpoint will be CNS ORR (NCT04303988).

The combination of immuno-oncology therapy using nivolumab (anti-PD-1, Phase 1 NCT03807765), atezolizumab (anti-PD-L1, Phase 2 NCT03483012) or pembrolizumab (anti-PD-1, Phase 1/2 NCT03449238) plus SRS is also under investigation. This latter trial is recruiting patients with at least two BM who are eligible to receive SRS in order to evaluate the potential abscopal effect of irradiating one lesion on other, non-irradiated lesions.

### Other targeted therapy

A genomic-guided Phase 2 trial (Alliance A071701, NCT03994796) in which biopsies of intracranial and extracranial lesions are performed on patients with progressive BM to allow targeted sequencing on specific pathways (neurotrophic tropomyosin receptor kinase (NTRK), ROS1 fusions, CDK and PI3K pathways) is ongoing. In the event of actionable mutations, a matched targeted therapeutic agent known to have CNS penetrance will be proposed (entrectinib for NTRK and ROS1, abemaciclib for CDKs or GDC-0084 for PI3K). The objective is to determine whether targeting specific BM mutations will improve clinical outcomes.

Several studies are ongoing to further analyse targeted therapies such as CDK4/6 inhibitors, TKIs, PI3K inhibitors and ATM inhibitors. CDK4/6 inhibitors, which are able to cross the BBB, have generated a lot of interest. As well as the study with abemaciclib,^89^ a single-arm Phase 2 pilot trial with palbociclib monotherapy is currently enrolling patients with HER2^+^ BCBM (ER^+^ and ER) (NCT02774681). Furthermore, a single-arm trial in China will evaluate the efficacy of combining palbociclib, trastuzumab and lapatinib with fulvestrant in ER^+^/HER2^+^ BCBM (NCT04334330). For patients with ‘triple positive’ MBC, another original combination of tucatinib, abemaciclib and trastuzumab is currently being tested, with specific CNS secondary endpoints (NCT03846583).

With regard to TKIs, afatinib, an inhibitor of HER2 and other EGFRs, is currently being analysed: a Phase 1 study will determine the appropriate dose and safety for patients with HER2^+^ BM (NCT02423525) and a Phase 2 study will analyse afatinib penetrance into BM for patients having undergone BM surgery, with or without low-dose targeted radiation (NCT02768337). Afatinib is also being tested in combination with T-DM1 vs T-DM1 alone in a Phase 1/2 study (HER2BAT NCT04158947). Pyrotinib, an inhibitor of HER1, HER2 and HER4, is being tested in combination with vinorelbine in a single-arm Phase 2 trial for women with HER2^+^ BM (NCT03933982). Pyrotinib is also to be tested in combination with temozolomide injection (NCT04303988 cohort HR+/HER2+) and in combination with capecitabine (NCT03691051) in two Phase 2 single-arm trials, with CNS ORR as the primary endpoint. The anti-angiogenic TKI sorafenib and AZD1390, an ATM inhibitor, are both being tested in combination with WBRT in Phase 1 clinical trials (NCT01724606 and NCT03423628). Finally, the safety and efficacy of the combination of the PI3K inhibitor GDC-0084 with trastuzumab is being trialled in a single-arm Phase 2 trial for HER2^+^ BM (NCT03765983).

### Intrathecal trastuzumab

Another emerging area of interest is the use of intrathecal trastuzumab for patients with leptomeningeal metastases. Numerous case reports and retrospective cohorts have compared intrathecal trastuzumab, intrathecal chemotherapy and WBRT, and shown intrathecal trastuzumab to be efficacious for some patients.^100,101^ The Phase 1 part of the study NCT01373710 has been published,^102^ and suggests a Phase 2 weekly dose of intrathecal trastuzumab of 150 mg. The Phase 2 trial using this dose regimen is ongoing. In another Phase 1 trial (NCT01325207), intrathecal trastuzumab was administrated twice a week for 1 month, then once a week for another month and then every week until progression.^103^ The maximum tolerated dose was 80 mg. In Phase 2, 5 patients (19.2%) showed partial response, 13 patients (50%) had stable disease and 8 patients (30.8%) showed disease progression. Median PFS was 2.4 months and median OS was 12.1 months. The primary endpoint of a 25% response rate has not yet been met. However, 69% of patients showed clinical benefit (stable disease or better).^103^

### High-dose trastuzumab

The use of high-dose trastuzumab (6 mg/w) with pertuzumab to increase the intracerebral trastuzumab concentration has also been tested in patients with progressive BM after radiotherapy.^104^ This study was based on the fact that preclinical data supported the dose-dependent activity of trastuzumab in intracranial tumour models.^32^ Lin et al. demonstrated a modest clinical benefit with a pertuzumab–high-dose-trastuzumab combination in a Phase 2, open-label, single-arm study of 40 patients; CNS 0RR was 11% and 6-month CBR was 51%. No excess cardiotoxicity was observed but there was an increased incidence of limited grade 1–2 diarrhoea. Another way to increase trastuzumab concentration is to use a super-selective intra-arterial cerebral infusion of trastuzumab. A trial using such an approach is underway in patients with HER2^+^ BCBM (NCT02571530).

### Blood–brain barrier disruption (BBBD)

**Fig. 2:**
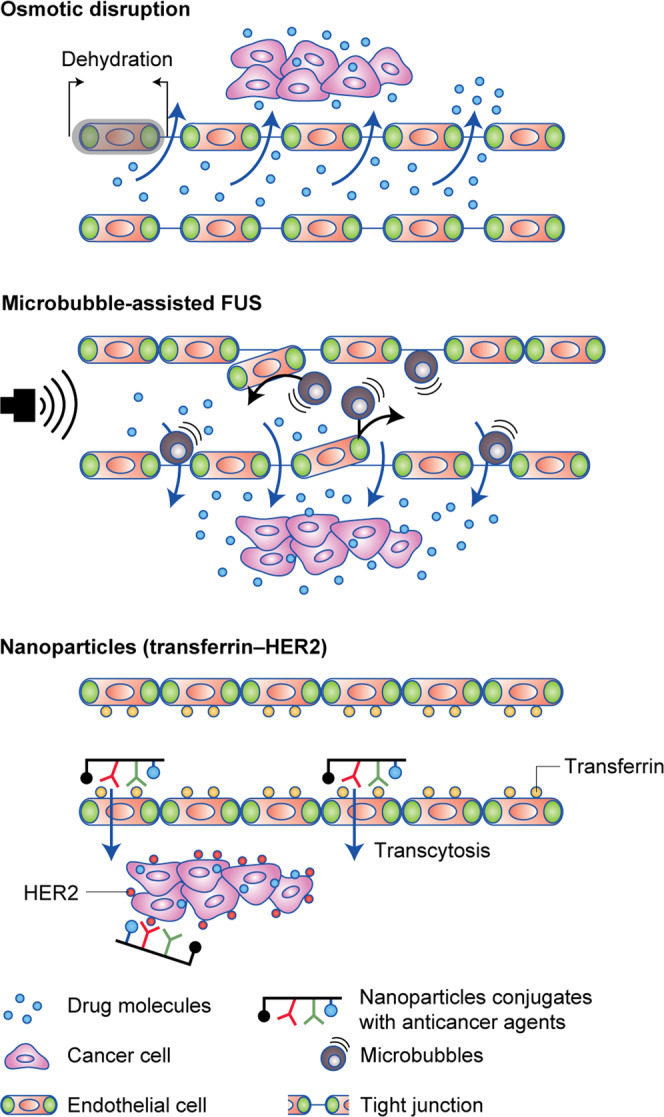
Main blood–brain barrier (BBB) disrupting strategies under development: osmotic disruption, microbubble-assisted focused ultrasound (FUS) and nanoparticules conjugated with cancer agent.

As the BBB could, in theory, hinder drug delivery to the brain, three different strategies—involving chemical and mechanical means—are being developed to transiently disrupt the BBB to deliver higher concentrations of anti-HER2 drugs to the brain (Fig. 2). Obviously, such strategies will be of interest in the clinical situation where the BBB is indeed effective, and future clinical trials will have to assess this point.^105^ The first approach uses osmotic BBBD with certain drugs such as mannitol to open the blood vessels around the brain to enable methotrexate and carboplatin with or without trastuzumab to penetrate. However, a clinical trial using this method was discontinued prematurely owing to lack of evidence of efficacy (NCT00397501). Other, more promising, strategies are currently still at the preclinical stage. One technology currently undergoing testing is microbubble-assisted focused ultrasound (FUS), which uses oscillating microbubbles to generate micrometre-scale mechanofluidic effects to enhance drug transport. The combination of trastuzumab and FUS showed anticancer activity in the rat brain: after six weekly trastuzumab treatments with FUS, the mean tumour volume was significantly reduced, and survival was significantly prolonged.^106^ The combination of trastuzumab, pertuzumab and FUS also delayed the progression of experimental brain metastases.^107^ The Exablate Neuro system (INSIGHTEC), which serially disrupts the BBB temporarily and locally with MRI-guided FUS, will be tested in patients with HER2^+^ BCBM (NCT03714243). The third technology tested involves the use of nanoparticles conjugated with anticancer agents. Patil et al. demonstrated that, compared with the phosphate-buffered saline control, tumour-targeted nanoconjugates carrying molecular inhibitors of EGFR or HER2 significantly prolonged the survival of mice with Her2^+^ BCBM.^108^ Hamilton et al. also showed that nanoparticles coated with a tumour-penetrating peptide (iRGD) strongly inhibited tumour progression when applied in the early stages of metastasis development, indicating that this technology might provide a promising treatment for the prevention of BM.^109^

## Systematic screening for the detection of BM?

Due to the lack of data demonstrating a clinical benefit, brain screening for patients with MBC is not currently recommended in the US NCCN and ESMO guidelines.^23^ Nevertheless, patients at high risk of developing BM could potentially benefit from screening strategies, as an earlier diagnosis could lead to a reduction in WBRT use and enable localised, less toxic and more effective BM treatment in a higher proportion of cases.^23,110,111^ Four studies are exploring the value of systematic radiological screening.

In the SYMPToM trial (NCT03881605), 50 women with HER2^+^ or triple-negative MBC will be randomised to receive either MRI or clinical surveillance for BM every 4 months for 1 year, although any patient under clinical surveillance who develops symptoms will receive an MRI. The Dana-Farber Cancer Institute trial (NCT04030507) will contain four patient cohorts: patients with triple-negative breast cancer will undergo screening MRI of the brain (single arm, one cohort), patients with ER^+^/HER2^–^ and HER2^+^ subtypes will be randomised between screening MRI of the brain or not (two cohorts) and patients with inflammatory BC will undergo screening MRI of the brain (one cohort). The primary endpoints will be quality of life at 12 months, incidence of symptomatic BM and incidence of BM. Another trial, NCT03617341, comprises an observational cohort of 200 patients with TNBC or HER2^+^ BC in which MRI of the brain will be undertaken at the time of initial diagnosis, first- and second-line treatment failure. Finally, a randomised trial will analyse the survival outcome (without neurological symptoms owing to BM) in patients with HER^+^ MBC who undergo a brain MRI once every 4 months vs once every 12 months (NCT00398437).

## Conclusions

The increased survival rates of breast cancer patients who have metastatic disease mean that an increasing incidence of BCBM, alongside the associated impairment of quality of life and OS, has become a problem that requires improved solutions. Local treatment, with surgery when possible, and SRS, remains central in the current treatment strategy. With regard to medical treatment, multiple studies have shown that the results obtained with conventional chemotherapy and targeted therapies are mostly consistent with the systemic efficacy observed with these same molecules. Thus, the intrinsic efficacy on tumour type appears to be more important than the issue of BBB diffusion to predict treatment usefulness, owing to the fact that the BTB is more permeable to systemic molecules than the intact BBB. Nevertheless, when patients with progressive BM after radiation therapy are treated with systemic treatment, prognosis remains poor, which has been recently suggested to correlate with additional organ-specific genomic alterations.^13^ The best results published to date have been obtained in patients with BM from HER2^+^ MBC, for whom all currently used combinations of chemotherapy and anti-HER2 therapy have shown some efficacy, with particularly impressive results obtained with the tucatinib–trastuzumab–capecitabine combination. Patients with BM from luminal or triple-negative MBC have fewer medical options currently, but CDK inhibitors, PARP inhibitors, PI3K inhibitors and immuno-oncology therapy are promising therapeutic candidates.

The individuality of patients with BCBM, in terms of clinical characteristics and treatment resistance, makes it necessary to develop specific clinical trials to generate new treatment strategies.^105^ In the near future, brain-specific therapies that target crosstalk with the microenvironment and brain-specific genomic alterations could provide significant benefits for patients. New therapeutic strategies with combinations of immunotherapy, radiotherapy and targeted therapies could increase efficacy while limiting the side effects. Finally, preventing the development of BM in the adjuvant setting using molecules that can cross the intact BBB is a particularly attractive option.

## Data Availability

Not applicable.
